# Xanthohumol, a Prenylated Flavonoid from Hops (*Humulus lupulus*), Prevents Platelet Activation in Human Platelets

**DOI:** 10.1155/2012/852362

**Published:** 2012-05-07

**Authors:** Ye-Ming Lee, Kuo-Hsien Hsieh, Wan-Jung Lu, Hsiu-Chu Chou, Duen-Suey Chou, Li-Ming Lien, Joen-Rong Sheu, Kuan-Hung Lin

**Affiliations:** ^1^Department of Surgery, Hsinchu Mackay Memorial Hospital, Hsinchu and Mackay Medicine, Nursing and Management College, 92 Zhong-Shan N. Road, Taipei 104, Taiwan; ^2^Department of Pharmacology and Graduate Institute of Medical Sciences, Taipei Medical University, 250 Wu-Hsing Street, Taipei 110, Taiwan; ^3^Department of Orthopedics, Tungs' Taichung MetroHarbor Hospital, 699 Chung-Chi Road, Taichung 435, Taiwan; ^4^Central Laboratory, Shin-Kong Wu Ho-Su Memorial Hospital, 95 Wen-Chang Road, Taipei 111, Taiwan

## Abstract

Xanthohumol is the principal prenylated flavonoid in the hop plant (*Humulus lupulus* L.). Xanthohumol was found to be a very potent cancer chemopreventive agent through regulation of diverse mechanisms. However, no data are available concerning the effects of xanthohumol on platelet activation. The aim of this paper was to examine the antiplatelet effect of xanthohumol in washed human platelets. In the present paper, xanthohumol exhibited more-potent activity in inhibiting platelet aggregation stimulated by collagen. Xanthohumol inhibited platelet activation accompanied by relative [Ca^2+^]*_i_* mobilization, thromboxane A_2_ formation, hydroxyl radical (OH^●^) formation, and phospholipase C (PLC)**γ**2, protein kinase C (PKC), mitogen-activated protein kinase (MAPK), and Akt phosphorylation. Neither SQ22536, an inhibitor of adenylate cyclase, nor ODQ, an inhibitor of guanylate cyclase, reversed the xanthohumol-mediated inhibitory effect on platelet aggregation. Furthermore, xanthohumol did not significantly increase nitrate formation in platelets. This study demonstrates for the first time that xanthohumol possesses potent antiplatelet activity which may initially inhibit the PI3-kinase/Akt, p38 MAPK, and PLC**γ**2-PKC cascades, followed by inhibition of the thromboxane A_2_ formation, thereby leading to inhibition of [Ca^2+^]*_i_* and finally inhibition of platelet aggregation. Therefore, this novel role of xanthohumol may represent a high therapeutic potential for treatment or prevention of cardiovascular diseases.

## 1. Introduction

Dietary factors play key roles in developing and preventing various human diseases, including cardiovascular diseases (CVDs). Epidemiological studies showed an inverse relationship between diets rich in fruits, vegetables, and spices, and the risk of all causes of death from cancer and CVD [1]. Hops (*Humulus lupulus* L.) have long been used in the brewing industry as a preservative and flavoring agent to add bitterness and aroma to beer [2]. In traditional Chinese medicine, hops are used to treat insomnia, restlessness, dyspepsia, and lack of an appetite. Alcoholic extracts of hops are clinically used in China to treat leprosy, pulmonary tuberculosis, acute bacterial dysentery, silicosis, and asbestosis with positive outcomes [2]. Xanthohumol is the principal prenylated flavonoid in the hop plant. Recently, xanthohumol has attracted considerable interest because of its biological activities, including anticancer, antiangiogenesis, anti-inflammation, and antioxidation [3]. Xanthohumol (1~50 *μ*M) was shown to suppress tumor growth by inhibiting cell proliferation and inducing apoptosis in various carcinoma cells [4–6]. It also exhibited antiangiogenic activity through inhibiting nuclear factor (NF)-*κ*B and Akt activation in vascular endothelial cells [7]. Furthermore, xanthohumol was reported to regulate the function and survival of immune cells by inhibiting the production of two important cytokines, monocyte chemoattractant protein (MCP)-1, and tumor necrosis factor (TNF)-*α*, in lipopolysaccharide-stimulated macrophages [8]. Xanthohumol also prevented hepatic inflammation and fibrosis *in vivo* and promoted dendritic cell apoptosis [3, 9]. Furthermore, xanthohumol was able to scavenge reactive oxygen species (ROS) in tissue-plasminogen activator-(TPA-) stimulated differentiated HL-60 cells [4].

Intravascular thrombosis is one of the generators of a wide variety of CVDs. Initiation of an intraluminal thrombosis is believed to involve platelet adherence and aggregation. Thus, platelet aggregation may play a crucial role in the atherothrombotic process [10]. Blood platelet activation and aggregation are common denominators in atherothrombotic events. Platelets are exclusively viewed as mediators of thrombosis and hemostasis. Therefore, investigation of the use of antiplatelet agents that inhibit atherothrombotic events (myocardial infarction, ischemic stroke, and vascular death) is warranted.

 Olas et al. [11] reported that the extract of hops significantly reduced oxidative stress in peroxynitrite-stimulated platelets. However, the detailed mechanisms underlying xanthohumol's signaling pathways in regulating platelet functions remain obscure. In the present study, we therefore for the first time examined in detail cellular signaling events associated with xanthohumol-mediated platelet function.

## 2. Materials and Methods

### 2.1. Materials

Xanthohumol, collagen (type I), luciferin-luciferase, arachidonic acid (AA), phorbol-12,13-dibutyrate (PDBu), 5,5-dimethyl-1 pyrroline N-oxide (DMPO), SQ22536, ODQ, and thrombin were purchased from Sigma (St. Louis, MO, USA). Fura 2-AM and fluorescein isothiocyanate (FITC) were from Molecular Probe (Eugene, OR, USA). The thromboxane B_2_ enzyme immunoassay (EIA) kit was from Cayman (Ann Arbor, MI, USA). The anti-phospho-p38 mitogen-activated protein kinase (MAPK) Ser^182^ monoclonal antibody (mAb) was from Santa Cruz (Santa Cruz, CA). The anti-p38 MAPK and anti-phospho-c-Jun N-terminal kinase (JNK) (Thr^183^/Tyr^185^) mAbs, and antiphospholipase C*γ*2 (PLC*γ*2), anti-phospho (Tyr^759^) PLC*γ*2, and anti-phospho-p44/p42 extracellular signal-regulated kinase (ERK) (Thr^202^/Tyr^204^) polyclonal antibodies (pAbs) were from Cell Signaling (Beverly, MA, USA). Anti-phospho-Akt (Ser^473^) and anti-Akt mAbs were from Biovision (Mountain View, CA, USA). The anti-*α*-tubulin mAb was from NeoMarkers (Fremont, CA, USA). The Hybond-P polyvinylidene difluoride (PVDF) membrane, enhanced chemiluminescence (ECL) western blotting detection reagent, and analysis system, horseradish peroxidase-(HRP-) conjugated donkey anti-rabbit immunoglobulin G (IgG), and sheep anti-mouse IgG were from Amersham (Buckinghamshire, UK). Xanthohumol was dissolved in 0.5% dimethyl sulfoxide (DMSO) and stored at 4°C until used.

### 2.2. Platelet Aggregation

Human platelet suspensions were prepared as previously described [10]. This study was approved by the Institutional Review Board of  Taipei Medical University and conformed to the principles outlined in the *Helsinki Declaration*, and all human volunteers provided informed consent. In brief, blood was collected from healthy human volunteers who had taken no medication during the preceding 2 weeks, and was mixed with acid-citrate-dextrose solution. After centrifugation, the supernatant (platelet-rich plasma; PRP) was supplemented with 0.5 *μ*M prostaglandin E_1_ (PGE_1_) and 6.4 I U/mL heparin. Washed platelets were finally suspended in Tyrode's solution containing 3.5 mg/mL bovine serum albumin (BSA). The final concentration of Ca^2+^ in Tyrode's solution was 1 mM.

 A turbidimetric method was used to measure platelet aggregation [10], with a Lumi-Aggregometer (Payton, Scarborough, ON, Canada). Platelet suspensions (3.6 × 10^8^ cells/mL) were preincubated with various concentrations of xanthohumol or an isovolumetric solvent control (final concentration, 0.5% DMSO) for 3 min before the addition of agonists. The reaction was allowed to proceed for 6 min, and the extent of aggregation was expressed in light-transmission units. When measuring ATP release, 20 *μ*L of a luciferin/luciferase mixture was added 1 min before the addition of agonists, and ATP release was compared to that of the control.

### 2.3. Flow Cytometric Analysis

Fluorescence-conjugated triflavin, an *α*_IIb_ *β*_3_ disintegrin, was prepared as previously described [12]. Platelet suspensions (3.6 × 10^8^ cells/mL) were preincubated with 1.5 and 3 *μ*M xanthohumol or a solvent control for 3 min, followed by the addition of 2 *μ*L of 2 *μ*g/mL FITC-triflavin. Suspensions were then assayed for fluorescein-labeled platelets using a flow cytometer (Beckman Coulter, Miami, FL, USA). Data were collected from 50,000 platelets per experimental group, and platelets were identified on the basis of their characteristic forward and orthogonal light-scattering profiles. All experiments were repeated at least four times to ensure reproducibility.

### 2.4. Measurement of Relative [Ca^2+^]_i_ Mobilization by Fura 2-AM Fluorescence

Citrated whole blood was centrifuged at 120 × g for 10 min. The supernatant was incubated with 5 *μ*M Fura 2-AM for 1 h. Human platelets were then prepared as described above. Finally, the external Ca^2+^ concentration of the platelet suspensions was adjusted to 1 mM. Relative [Ca^2+^]_i_ mobilization was measured using a fluorescence spectrophotometer (CAF 110, Jasco, Tokyo, Japan) with excitation wavelengths of 340 and 380 nm, and an emission wavelength of 500 nm [10].

### 2.5. Measurement of Thromboxane B_2_ Formation

Platelet suspensions (3.6 × 10^8^ cells/mL) were preincubated with xanthohumol (1.5~10 *μ*M) or a solvent control for 3 min before the addition of agonists. Six minutes after the addition of agonists, 2 mM EDTA and 50 *μ*M indomethacin were added to the suspensions. Thromboxane B_2_ levels of the supernatants were measured using an EIA kit.

### 2.6. Immunoblotting

Washed platelets (1.2 × 10^9^ cells/mL) were preincubated with 1.5 and 3 *μ*M xanthohumol or a solvent control for 3 min, followed by the addition of agonists to trigger platelet activation. The reaction was stopped, and platelets were immediately resuspended in 200 *μ*L of lysis buffer. Samples containing 80 *μ*g of protein were separated by a 12% sodium dodecylsulfate polyacrylamide gel electrophoresis (SDS-PAGE); proteins were electrotransferred by semidry transfer (Bio-Rad, Hercules, CA, USA). Blots were blocked with TBST (10 mM Tris-base, 100 mM NaCl, and 0.01% Tween 20) containing 5% BSA for 1 h and then probed with various primary antibodies. Membranes were incubated with HRP-linked anti-mouse IgG or anti-rabbit IgG (diluted 1 : 3000 in TBST) for 1 h. Immunoreactive bands were detected by an ECL system. The bar graph depicts the ratios of semiquantitative results obtained by scanning reactive bands and quantifying the optical density using videodensitometry (Bio-profil; Biolight Windows Application V2000.01; Vilber Lourmat, France).

### 2.7. Estimation of Nitrate Formation

In brief, platelet suspensions (10^9^ cells/mL) were preincubated with 1.5 and 3 *μ*M xanthohumol or a solvent control for 3 min, followed by centrifugation. The amount of nitrate in the platelet suspensions (10 *μ*L) was measured by adding a reducing agent to the purge vessel to convert nitrate to NO which was stripped from the suspensions by purging with helium gas [12]. The NO was then drawn into a Sievers Nitric Oxide Analyzer (Sievers 280 NOA, Boulder, CO, USA). Nitrate concentrations were calculated by comparison to standard solutions of sodium nitrate.

### 2.8. Measurement of Hydroxyl Radicals by Electron Spin Resonance (ESR) Spectrometry

The ESR method used a Bruker EMX ESR spectrometer as described previously [13]. In brief, platelet suspensions (3.6 × 10^8^ cells/mL) were preincubated with 1.5 and 3 *μ*M xanthohumol or a solvent control for 3 min before the addition of 1 *μ*g/mL collagen. The reaction was allowed to proceed for 5 min, followed by the addition of 100 *μ*M DMPO for the ESR study. The rate of free radical-scavenging activity was defined by the following equation:

(1) $$\text{inhibition rate}\;=\;1\;\text{–}\;\left[\frac{\text{signal height (xanthohumol)}}{\text{signal height (control)}}\right],$$

(see [13]).

### 2.9. Data Analysis

Experimental results are expressed as the means ± S.E.M. and are accompanied by the number of observations. Experiments were assessed by an analysis of variance (ANOVA). If this analysis indicated significant differences among group means, then each group was compared using the Newman-Keuls method. *P* < 0.05 was considered statistically significant.

## 3. Results

### 3.1. Effects of Xanthohumol on Platelet Aggregation and Relative [Ca^2+^]_i_ Mobilization in Washed Human Platelets

**Figure 1:**
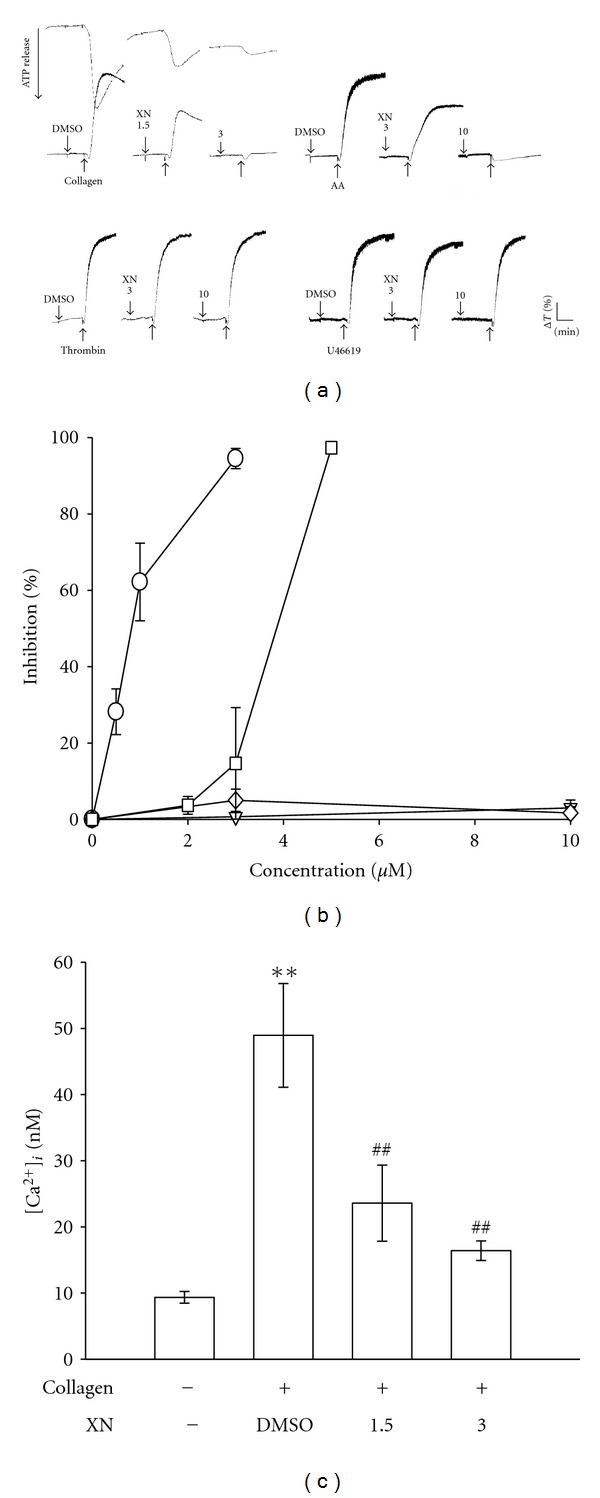
Effects of xanthohumol (XN) on the inhibition of platelet aggregation and relative [Ca^2+^]*_i_* mobilization in activated platelets. Washed platelets (3.6 × 10^8^ cells/mL) were preincubated with 1.5~10 *μ*M XN or a solvent control (0.5% DMSO), followed by the addition of 1 *μ*g/mL collagen (◯), 1 *μ*M U46619 (▿), 60 *μ*M arachidonic acid (AA; □), or 0.05 IU/mL thrombin (*◊*) to trigger ((a)-(b)) platelet aggregation and an ATP-release reaction ((a), top-left corner) or (c) relative [Ca^2+^]*_i_* mobilization. Profiles (a) are representative examples of six similar experiments. Data ((b) and (c)) are presented as the means ± S.E.M. ((b), *n* = 6; (c), *n* = 3); **P < 0.01, compared to the resting group; ^##^ *P* < 0.01, compared to the control (DMSO) group.

Xanthohumol (1.5 and 3 *μ*M) exhibited very potent activity in inhibiting platelet aggregation and the ATP-release reaction stimulated by 1 *μ*g/mL collagen (Figure 1(a)); it also significantly inhibited platelet aggregation stimulated by 60 *μ*M AA at higher concentrations (3 and 10 *μ*M) (Figure 1(a)). However, it did not significantly inhibit platelet aggregation stimulated by 0.05 U/mL thrombin or 1 *μ*M U46619, a prostaglandin endoperoxide, at the same concentrations (3 and 10 *μ*M) (Figure 1(a)). The 50% inhibitory concentration (IC_50_) value of xanthohumol for platelet aggregation induced by collagen was approximately 1.5 *μ*M (Figure 1(b)). The solvent control (0.5% DMSO) did not significantly affect platelet aggregation stimulated by agonists (Figure 1(a)). In subsequent experiments, we used collagen as an agonist to explore the inhibitory mechanisms of xanthohumol in platelet activation.

 As shown in Figure 1(c), 1 *μ*g/mL collagen evoked a marked increase in relative Ca^2+^ mobilization, and this increase was markedly inhibited in the presence of xanthohumol (1.5 *μ*M, 50.8 ± 9.5% and 3 *μ*M, 66.5 ± 9.6%; *n* = 3).

### 3.2. Influence of Xanthohumol on *α*_IIb_ *β*_3 _ Integrin Conformational Changes, Thromboxane B_2_ Formation, and PLC*γ*2 Phosphorylation

**Figure 2:**
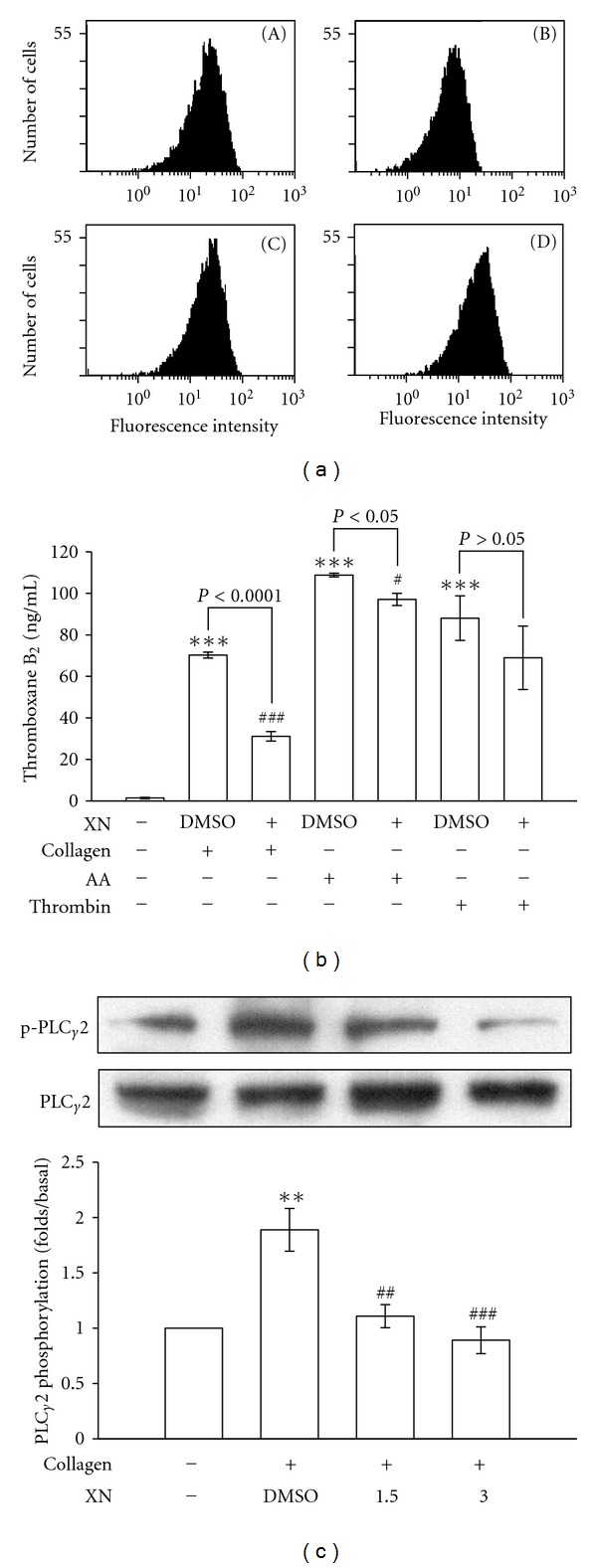
Effects of xanthohumol (XN) on FITC-triflavin binding to the *α*_IIb_ *β*_3_ integrin, thromboxane B_2_ formation, and phospholipase C*γ*2 (PLC*γ*2) phosphorylation in activated platelets. (a) The solid line represents the fluorescence profiles of (A) 2 *μ*g/mL FITC-triflavin in the absence of XN as a positive control, (B) in the presence of 5 mM EDTA as a negative control, or in the presence of (C) 1.5 and (D) 3 *μ*M XN, followed by the addition of 2 *μ*g/mL FITC-triflavin. For other experiments, washed platelets were preincubated with 3 *μ*M XN or 0.5% DMSO, followed by the addition of 1 *μ*g/mL collagen, 60 *μ*M arachidonic acid (AA), or 0.05 U/mL thrombin to trigger platelet activation. Cells were collected, and subcellular extracts were analyzed for (b) thromboxane B_2_ formation, and (c) PLC*γ*2 phosphorylation as described in Section 2. Profiles (a) are representative examples of five similar experiments. Data ((b) and (c)) are presented as the means ± S.E.M. ((b), *n* = 4; (c), *n* = 6). **P < 0.01 and ***P < 0.001, compared to the resting group; ^#^ *P* < 0.05, ^##^ *P* < 0.01 and ^ ###^ *P* < 0.001, compared to the control (DMSO) group.

Triflavin is an *α*_IIb_ *β*_3_ disintegrin which inhibits platelet aggregation by directly interfering with fibrinogen binding to the *α*_IIb_ *β*_3_ integrin [12]. Therefore, we further evaluated whether or not xanthohumol directly binds to the platelet *α*_IIb_ *β*_3_ integrin, leading to interruption of platelet aggregation. In this study, the relative intensity of the fluorescence of 2 *μ*g/mL FITC-triflavin bound directly to 1 *μ*g/mL collagen-activated platelets was 26.8 ± 0.5 (*n* = 5) (Figure 2(a) (A)), and it was markedly reduced in the presence of 5 mM EDTA (negative control, 9.7 ± 0.5, *P* < 0.001; *n* = 5) (Figure 2(a) (B)). Xanthohumol (1.5 and 3 *μ*M) did not significantly affect FITC-triflavin binding to the *α*_IIb_ *β*_3_ integrin in platelet suspensions (1.5 *μ*M, 26.9 ± 0.6; 3 *μ*M, 28.0 ± 0.7; *n* = 5) (Figure 2(a) (C, D)), indicating that the inhibitory effect of xanthohumol on platelet aggregation does not involve binding to the platelet *α*_IIb_ *β*_3_ integrin. Furthermore, resting platelets produced relatively low amounts of thromboxane B_2_ compared to agonist-activated platelets (i.e., collagen). At 3 *μ*M, xanthohumol inhibited both 1 *μ*g/mL collagen- and 60 *μ*M AA-stimulated thromboxane B_2_ formation by approximately 56% and 10%, respectively, but it was not significantly affected by 0.05 U/mL thrombin stimulation (Figure 2(b)). PLC hydrolyzes phosphatidylinositol 4,5-bisphosphate (PIP_2_) to generate two secondary messengers: inositol 1,4,5-trisphosphate (IP_3_) and diacylglycerol (DAG) [14]. DAG activates PKC, which then phosphorylates p47 proteins (p47). Treatment with 1.5 and 3 *μ*M xanthohumol concentration dependently abolished the phosphorylation of PLC*γ*2 stimulated by collagen (Figure 2(c)).

### 3.3. Xanthohumol on PKC Activation

**Figure 3:**
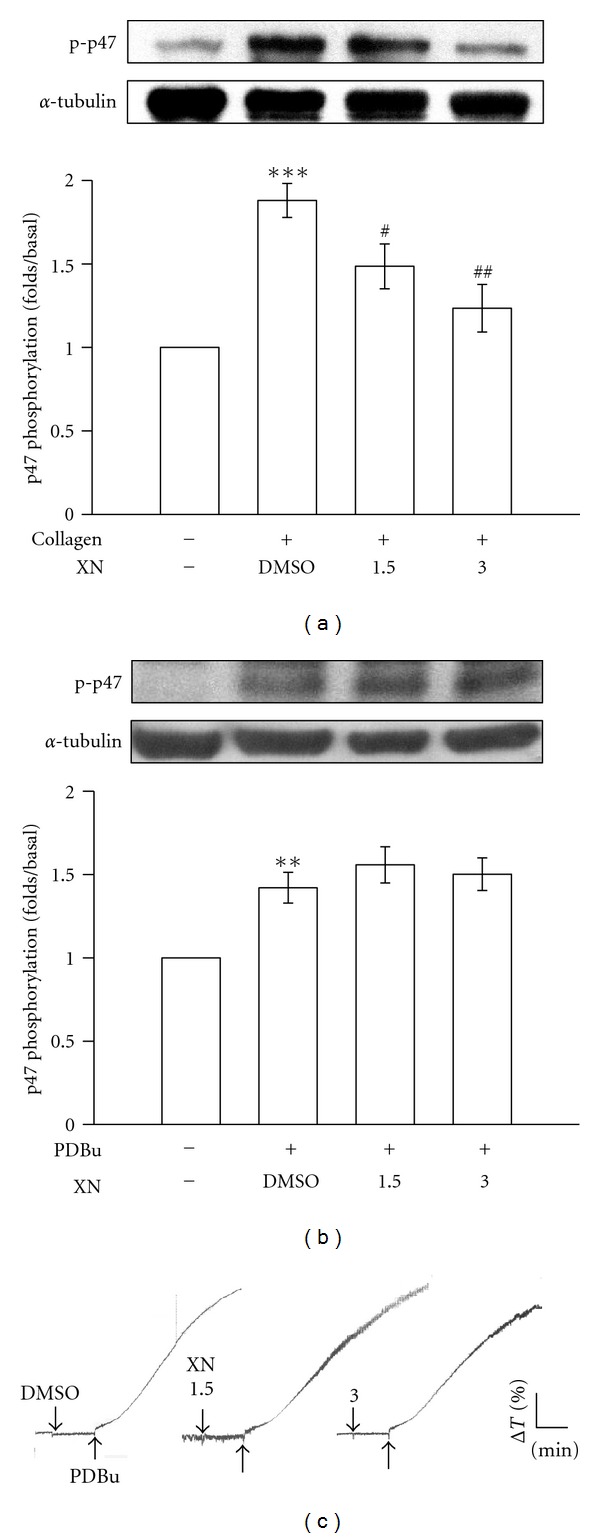
Inhibitory activity of xanthohumol (XN) on protein kinase C (PKC) activation in activated platelets. Washed platelets were preincubated with 1.5 and 3 *μ*M XN or 0.5% DMSO, followed by the addition of 1 *μ*g/mL collagen or 150 nM PDBu to trigger ((a) and (b)) PKC activation or (c) platelet aggregation. Cells were collected, and subcellular extracts were analyzed for ((a) and (b)) phosphorylation of the PKC substrate (p-p47) as described in Section 2. Data ((a) and (b)) are presented as the means ± S.E.M. (*n* = 5). **P < 0.01 and ***P < 0.001 compared to the resting group; ^#^ *P* < 0.05 and ^ ##^ *P* < 0.01 compared to the control (DMSO) group. Profiles (c) are representative examples of three similar experiments.

Stimulation of platelets with a number of different agonists (such as collagen) or PDBu, an activator of PKC [15], markedly induces PKC activation (p47 phosphorylation). In the present study, when 1 *μ*g/mL collagen (Figure 3(a)) or 150 nM PDBu (Figure 3(b)) was added to human platelets, a protein with an apparent molecular weight of 47 kDa (p47) was predominately phosphorylated compared to resting platelets. Xanthohumol (1.5 and 3 *μ*M) concentration dependently inhibited p47 phosphorylation stimulated by collagen but not by PDBu (Figures 3(a) and 3(b)). As shown in the Figure 3(c), xanthohumol did not significantly affect PDBu-(150 nM) induced platelet aggregation. These results indicate that xanthohumol abolished PKC activation through inhibiting PLC*γ*2 phosphorylation.

### 3.4. Xanthohumol Attenuates MAPKs and Akt Activation

**Figure 4:**
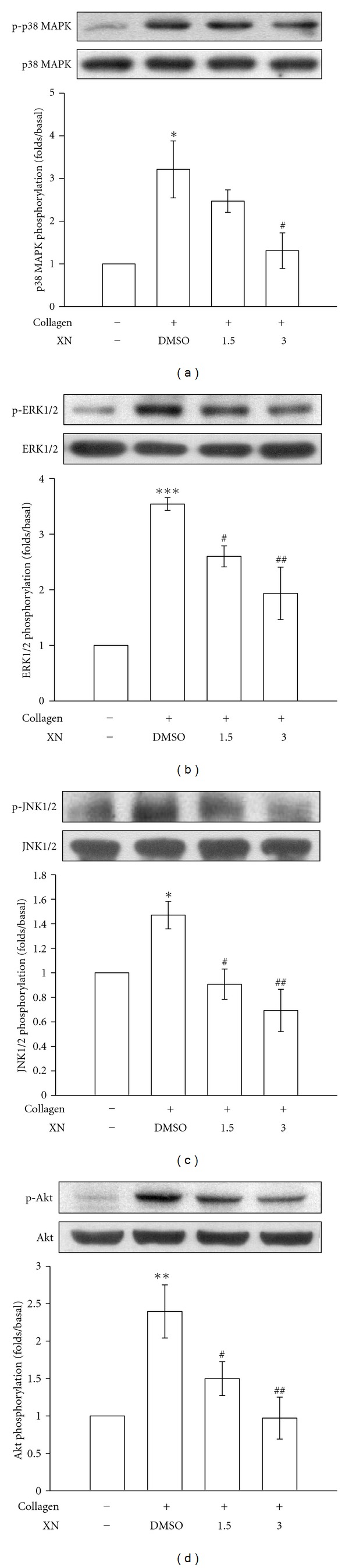
Xanthohumol (XN) on p38 MAPK, ERK1/2, JNK1/2, and Akt phosphorylation in collagen-activated platelets. Washed platelets (1.2 × 10^9^ cells/mL) were preincubated with 1.5 and 3 *μ*M XN or 0.5% DMSO, followed by the addition of 1 *μ*g/mL collagen to trigger platelet activation. Cells were collected, and subcellular extracts were analyzed for (a) p38 MAPK, (b) ERK1/2, (c) JNK1/2, and (d) Akt phosphorylation. Data are presented as the means ± S.E.M. (*n* = 4). *P < 0.05, **P < 0.01, and ***P < 0.001, compared to the resting group; ^#^ *P* < 0.05 and ^##^ *P* < 0.01, compared to the control (DMSO) group.

To further investigate the inhibitory mechanisms of xanthohumol in platelet activation, we detected several signaling molecules such as MAPKs (i.e., p38 MAPK, ERK1/2, and JNK1/2) and Akt. Xanthohumol (3 *μ*M) attenuated the phosphorylation of p38 MAPK (Figure 4(a)), ERK1/2 (Figure 4(b)), JNK1 (Figure 4(c)), and Akt (Figure 4(d)) stimulated by 1 *μ*g/mL collagen.

### 3.5. Effects of Xanthohumol on Cyclic Nucleotides, and Nitrate and Hydroxyl Radical (OH^●^) Formation in Collagen-Activated Platelets

**Figure 5:**
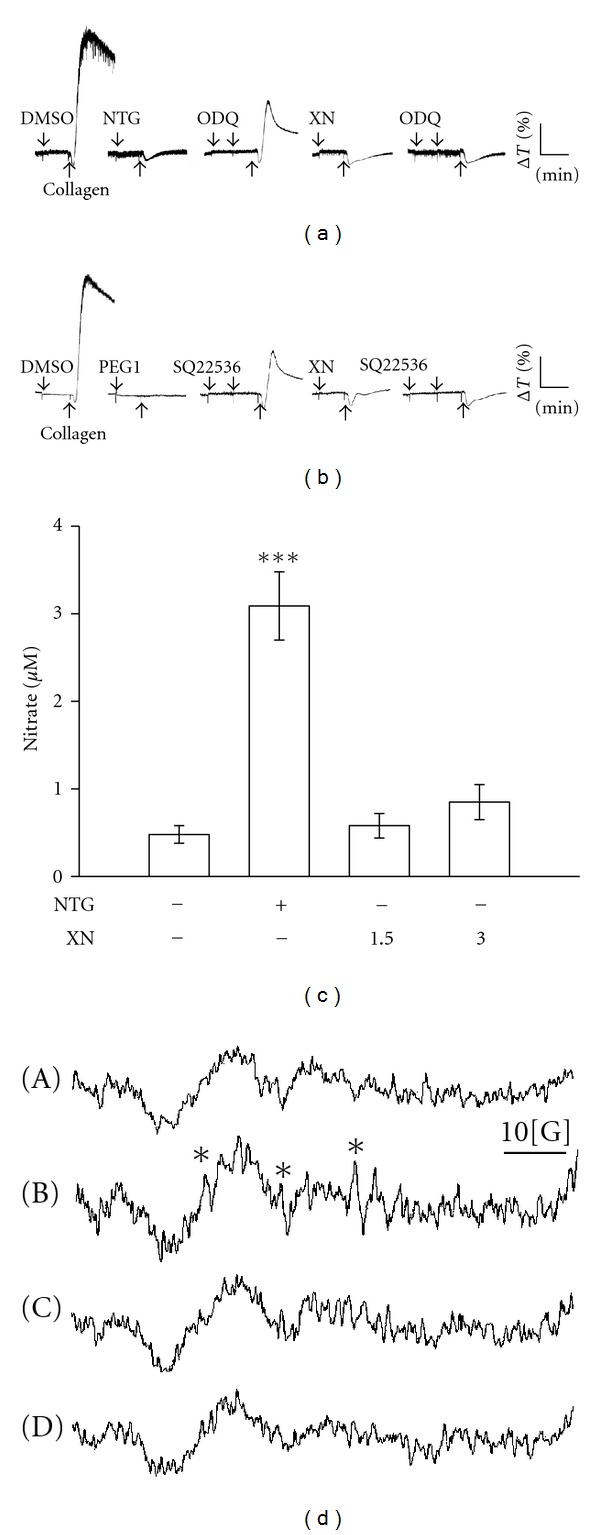
Regulation of platelet aggregation, nitrate formation, and hydroxyl radical (OH^●^) formation by xanthohumol (XN). ((a) and (b)) Washed platelets (3.6 × 10^8^ cells/mL) were preincubated with 10 *μ*M prostaglandin E_1_ (PGE_1_), 10 *μ*M nitroglycerin (NTG), or 3 *μ*M XN in the absence or presence of 20 *μ*M ODQ or 100 *μ*M SQ22536, followed by the addition of 1 *μ*g/mL collagen to trigger platelet aggregation. Profiles are representative examples of three similar experiments. On the other hand, (c) washed platelets (10^9^ cells/mL) were incubated with 10 *μ*M nitroglycerin (NTG), and 1.5 and 3 *μ*M XN. Cells were then collected, and subcellular extracts were analyzed for nitrate formation as described in Section 2. Data are presented as the means ± S.E.M. (*n* = 4). ***P < 0.001, compared to the resting group. (d) For the electron spin resonance (ESR) study, washed platelets (3.6 × 10^8^ cells/mL) were incubated with (A) Tyrode's solution only (resting group), or platelets were preincubated with (B) the control (0.5% DMSO), (C) 1.5 **μ**M, or (D) 3 *μ*M XN followed by the addition of 1 *μ*g/mL collagen to trigger hydroxyl radical formation. Spectra are representative examples of three similar experiments. An asterisk (*) indicates the formation of hydroxyl radicals.

As shown in Figures 5(a) and 5(b), ODQ (20 *μ*M) and SQ22536 (100 *μ*M), inhibitors of guanylate cyclase and adenylate cyclase, obviously reversed nitroglycerin-(NTG; 10 *μ*M) and PGE_1_-(10 *μ*M) mediated inhibition of platelet aggregation stimulated by collagen, respectively; however, neither inhibitor responded to xanthohumol (3 *μ*M)-mediated inhibition of platelet aggregation. Xanthohumol (3 *μ*M) did not significantly increase cyclic AMP or cyclic GMP levels in human platelets (data not shown). Furthermore, NO was quantified using a sensitive and specific ozone redox-chemiluminescence detector as shown in Figure 5(c). The production of nitrate markedly increased with 10 *μ*M NTG activation compared to the resting group, whereas xanthohumol (1.5 and 3 *μ*M) did not reach statistical significance. On the other hand, a typical ESR signal of hydroxyl radical (OH^●^) formation was triggered in collagen-activated platelets compared to resting platelets (Figure 5(d) (A, B)), and treatment with xanthohumol (1.5 and 3 *μ*M) obviously reduced hydroxyl radical formation stimulated by collagen (Figure 5(d) (C, D)).

## 4. Discussion

This study reveals for the first time that xanthohumol, besides its well-known anticancer properties, also possesses potent antiplatelet activity. Xanthohumol is the major prenylflavonoid of hops (0.1% ~ 1% on a dry weight basis) [16], and xanthohumol and its related prenylflavonoids are known to be contained in beer as a dietary source. The average person in the USA consumed 225 mL/day of beer in 2001 [16]. Therefore, the daily intake of total prenylflavonoids would be approximately 0.14 mg. Studies of the pharmacokinetics revealed that the maximal plasma concentration was approximately 180 nM after 4 h of oral administration of 50 mg/kg xanthohumol in rats [16]. Indeed, dietary intake of prenylflavonoids (xanthohumol) through normal beer consumption would not be sufficient to achieve plasma concentrations that could inhibit platelet activation. Ideally, atherothrombotic prevention is achieved by long-term exposure to nontoxic agents, preferably as part of certain food products or nutritional supplements. Xanthohumol showed no toxicity to liver cells nor did it inhibit mitochondrial respiration or uncouple oxidative phosphorylation in isolated rat liver mitochondria at 10 *μ*M [17]. Therefore, xanthohumol is considered to be the best phytochemical isolated from hops to investigate antiplatelet functions.

Gerhauser et al. [4] reported that xanthohumol can be an effective anti-inflammatory agent by inhibiting endogenous prostaglandin synthesis through inhibition of cyclooxygenases (constitutive COX-1 and inducible COX-2). In the present study, collagen-induced thromboxane B_2_ formation, a stable metabolite of thromboxane A_2_, was also markedly inhibited by xanthohumol. In platelets, AA is released from cell membranes and converted to thromboxane A_2_. Thromboxane A_2_ is important for collagen- and AA-induced platelet aggregation, which may explain the more-potent activity of xanthohumol in inhibiting collagen- and AA-induced platelet aggregation than other agonists (i.e., thrombin). Stimulation of platelets by agonists (i.e., collagen) causes marked alterations in phospholipid metabolism. Activation of PLC results in the production of IP_3_ and DAG, which activates PKC, inducing protein phosphorylation (p47) [18]. PKC activation represents a strategy adopted by cells to allow selected responses to specific activating signals in distinct cellular compartments [19]. PLC*γ*2 is involved in collagen-dependent signaling in platelets [20]. In this study, both PLC*γ*2 phosphorylation and PKC activation were inhibited by xanthohumol, suggesting that xanthohumol-mediated antiplatelet activity is involved in inhibiting the PLC*γ*2-PKC signal pathway.

 MAPKs consist of three major subgroups. ERKs (p44 ERK1 and p42 ERK2) are involved in proliferation, adhesion, and cell progression [21]. p38 MAPK and JNKs, which include the 46-kDa JNK1 and 55-kDa JNK2 isoforms, are involved in apoptosis [21]. ERK2, JNK1, and p38 MAPK were all identified in platelets [21]. The physiopathological roles of JNK1/2 and ERK1/2 are unclear in platelets, but they were suggested to be suppressors of *α*_IIb_ *β*_3_ integrin activation or negative regulators of platelet activation [22]. On the other hand, p38 MAPK provides a crucial signal for aggregation caused by collagen. We found that SB203580, an inhibitor of p38MAPK, markedly inhibited 1 *μ*g/mL collagen-induced platelet aggregation (data not shown). Among the numerous downstream targets of p38 MAPK, the most physiologically relevant one in platelets is cytosolic phospholipase A_2_ (cPLA_2_) which catalyzes AA release to produce thromboxane A_2_ [23]; thus, MAPKs (especially p38 MAPK) appear to have a pivotal role in platelet activation. On the other hand, knockout of Akt in mice resulted in defects of platelet activation stimulated by agonists [24, 25]. Akt functions as one of several downstream effectors of PI3-kinase [26]. Recently, we found that both p38 MAPK and PI3-kinase/Akt act in mutual activation as upstream regulators of PKC in activated platelets [27]. Therefore, Akt phosphorylation seems to play a crucial role in platelet activation. On the other hand, activation of human platelets is inhibited by two intracellular pathways regulated by either cyclic AMP or cyclic GMP. The importance of cyclic nucleotides in modulating platelet reactivity is well established [28]. In addition to inhibiting most platelet responses, elevated levels of cyclic nucleotides decrease intracellular Ca^2+^ concentrations by the uptake of Ca^2+^ into the dense tubular system (DTS) which negatively affects the actions of PLC and PKC [28]. Therefore, cyclic AMP and cyclic GMP act synergistically to inhibit platelet aggregation. In addition, platelets produce NO in smaller amounts than do endothelial cells [29]. Most cellular actions of NO occur via stimulation of intracellular guanylate cyclase, leading to increases in cyclic GMP. In this study, we found that xanthohumol-mediated antiplatelet activity, at least in part, was not regulated by NO or cyclic nucleotides.

 Reactive oxygen species (ROS; hydrogen peroxide, hydroxyl radicals, etc.) derived from platelet activation might amplify platelet reactivity during *in vivo *thrombus formation. Free radical species act as secondary messengers that increase cytosolic Ca^2+^ during the initial phase of platelet activation processes, and PKC is involved in receptor-mediated free radical production in platelets [12]. It is also evident that some of the hydrogen peroxide produced by platelets is converted into hydroxyl radicals, as platelet aggregation can be inhibited by hydroxyl radical scavengers [12]. ROS-scavenging activity of xanthohumol was studied by Gerhauser et al. [4] who found that xanthohumol was about 9-fold more potent than trolox at a concentration of 1 *μ*M at scavenging hydroxyl and peroxyl radicals as analyzed by an indirect method of an oxygen radical antioxidant capacity (ORAC) assay. In the present study, a similar result, obtained from the ESR study in which xanthohumol scavenges OH^●^ formation, provided direct evidence of its free radical-scavenging activity.

 Data generated in this investigation suggest that xanthohumol has a novel role in antiplatelet activation and can likely be used as a nutritional or dietary supplement as a prophylactic. Generally, a nutritional or dietary supplement is required to demonstrate a prophylactic effect in humans which may depend on individual characteristics; hence it may be impossible to delineate a selection of doses for time-course treatment, since it may vary from one individual to another. Nevertheless, this study provides new insights describing the mechanisms of xanthohumol at the studied doses in blocking specific signaling events during agonist-induced platelet activation.

 In conclusion, the most important findings of this study demonstrate for the first time that the very potent antiplatelet activity of xanthohumol may initially inhibit the PI3-kinase/Akt, p38 MAPK and PLC*γ*2-PKC cascades, followed by inhibition of thromboxane A_2_ formation, thereby leading to inhibition of [Ca^2+^]*_i_* and finally inhibition of platelet aggregation. Platelet aggregation plays important pathophysiological roles in a variety of thromboembolic disorders. Therefore, the novel role of xanthohumol in antiplatelet activation may represent high therapeutic potential for treating or preventing such diseases, in addition to it originally being considered as a chemopreventive agent.
